# Metabolic and hormonal effects of an 8 days water only fasting combined with exercise in middle aged men

**DOI:** 10.1038/s41598-025-05164-0

**Published:** 2025-07-02

**Authors:** Karol Pilis, Urszula Godlewska, Anna Pilis, Krzysztof Stec, Patrycja Dolibog, Marek Kruszewski, Adrianna Kosior-Lara, Romanbek Kalmatov, Wiesław Pilis, Sławomir Letkiewicz

**Affiliations:** 1 Department of Medical Science and Physiotherapy, Collegium Medicum, Jan Dlugosz University in Czestochowa, Waszyngtona 4/8, 42-200 Czestochowa, Poland; 2 Department of Medical Biophysics, Medical University of Silesia, 40-752 Katowice, Poland; 3 Department of Individual Sports, Jozef Pilsudski University of Physical Education in Warsaw, 00-968 Warsaw, Poland; 4 Medical Institute, Osh State University, 723500 Osh City, Kyrgyz Republic

**Keywords:** Fasting, Metabolism, Hormonal changes, Immunity, Physical activity, Metabolism, Endocrine system and metabolic diseases, Nutrition

## Abstract

Optimal nutrition and physical activity are vital for enhancing physical performance and preventing metabolic diseases like obesity. Recently, interest has grown in long-term fasting, particularly water-only fasting, which involves no food intake and unlimited water consumption. Our study investigated the effects of 8 days of water-only fasting combined with aerobic exercise in 13 middle-aged men, focusing on metabolic, hormonal, and immune changes. Results showed that fasting had a more significant impact than exercise, leading to changes in glucose, uric acid, IGF-1, IGF-2, GH, leptin, and cortisol, improved total antioxidant status (TAS), and reduced lipid peroxidation. While exercise enhanced the effects of fasting on triglycerides, insulin, GH, TAS, PerOX, and IL-6, changes in total protein and lactate were solely due to exercise. Overall, combining fasting and exercise led to a metabolic shift from carbohydrates to fatty acids and hormonal adaptations to stress. These results, although derived from a small group of patients, offer a promising outlook for further research into the effects of fasting combined with physical activity on health and weight maintenance.

## Introduction

Complete fasting, called a zero-calorie diet, involves completely stopping food intake while consuming any amount of water. The existing literature indicates both positive and negative aspects of prolonged fasting. However, it has not yet been clearly established how long a person should fast in order to secure optimal health benefits safely. In general, fasting unfolds through three distinct phases, each playing a vital role in the body’s adaptation: the post-absorptive phase (0–24 h), the gluconeogenic phase (24 h to 10 days), and the conservation of protein phase (beyond 10 days). Among them, the gluconeogenic phase stands out, during which the body changes the way it produces energy in the absence of external food sources^1^. To determine the optimal duration of fasting for health, the concept of hormetic adaptation was introduced. This concept aims to determine the moment during fasting when beneficial changes in the body cease and the possibility of deterioration of health begins^2^. The main feature of the hormetic concept is that low levels of biological, chemical, physical, or mental stress enhance adaptive responses that not only condition, repair, and restore normal functions of damaged tissues/organs, but moderately compensate, reducing ongoing underlying damage^3^.

It has been shown that a beneficial adaptation to fasting is, among others, reduced insulin concentration, which is a strong inhibitor of lipolysis^4,5^. Under these conditions, it was possible to release increased amounts of fatty acids and activate ketogenesis. An important point to note is that ketone bodies are used for energy production in most tissues of the body, except for erythrocytes and certain types of cancer cells. This is significant because it increases the survival rate of those tissues which are able to use ketone bodies during periods of fasting. It is worth mentioning that, after a few weeks of fasting, β-hydroxybutyrate and acetoacetate provide up to two thirds of the energy required by the brain^6^. During the initial stages of fasting, ketone bodies are associated with an increased release of free radical oxygen species (ROS) and elevated ratios of NAD+/NADH and AMP/ATP in mitochondria^7,8^. This initiates a temporary stress to the mitochondria, which activates a protective cellular response that subsequently leads to an improvement in mitochondrial function and the body’s ability to combat oxidative stress^8^. During fasting, the consumption of ketone bodies increases also in the heart^9^ and in skeletal muscles^10^. According to a study by Evans et al.^11^ the use of ketone bodies during intense exercise may increase by up to five times. However, higher concentrations of β-hydroxybutyrate can induce oxidative stress, typically by causing inflammation that damages lipids, proteins, and DNA in cells, as a result of the production of pro-inflammatory cytokines, tumor necrosis factor-α (TNFα), interleukins IL-1β and IL-6, or the chemokine CCL2^12,13^. Apart from this particular situation, different fasting models demonstrate that caloric restriction can positively regulate immune response and speed up regeneration processes such as angiogenesis or wound healing^14,15^. Furthermore, during physical activity, skeletal muscle plays a significant role as a secretory organ by releasing various myokines, including IL-6, which helps regulate energy metabolism^16^. Similarly, TNF-α has widespread metabolic effects throughout the body, and both cytokine concentrations can vary during certain conditions such as fasting and exercise^17–19^. It should be added that physical exercise, just like fasting, can be a very powerful agent that changes the body’s metabolism in many directions, depending on its intensity and duration.

It has been shown that controlled physical exercise included in the training framework resulted in, among others, positive regulation of the concentrations of adipokines (lipocain 2, omentin 1 or neuralgin 4) and many myokines (decortin, follistatin, myostatin, activin A, transforming growth factor beta-1—TGF-B-1) and beneficial cardiometabolic and somatic changes in obese men^20–22^.

The regulation of carbohydrate and lipid metabolism is critically influenced by hormones, cytokines, and myokines. Moreover, physical activity plays a pivotal role in enhancing metabolic processes, leading to substantial health benefits. Despite this, our understanding of how fasting, when paired with physical activity, affects metabolic regulation remains limited. It is essential to explore whether this combination produces beneficial or adverse effects. To address this important question, we will conduct a examination of the impacts of fasting and physical activity on metabolic parameters, pro-inflammatory markers, and oxidative stress. This study is a first step in uncovering the mechanisms by which water-based fasting, when paired with exercise, can enhance human health and overall well-being.

### Purpose of the study

The paper aims to demonstrate the positive and negative effects of the simultaneous impact of 8-days of water-only fasting and two maximum intensity exercise tests on the bodies of healthy, middle-aged men. This assessment has been made by recording the metabolic, hormonal, and immune changes in the body modified by both those factors.

## Material and methods

### Participants

Thirteen healthy, middle-aged men (57.25 ± 9.93 years; 81.72 ± 9.19 kg; 178.54 ± 4.77 cm) volunteered to undergo 8-days of water-only fasting, during which each individual consumed ad libitum moderately mineralized water of an identical composition. The research participants were selected from an advertisement in the press. The conditions for initial qualification were male gender, age between 30 and 70 years, good health, previous personal experience in fasting for several days, average physical activity, following a mixed diet, not using alcohol, nicotine, psychoactive substances and excess medications. The body weight of the participants was 60–95 kg, body mass index 20–29.9 kg/m^2^, systolic blood pressure 100–140 mmHg and diastolic blood pressure 60–90 mmHg. Medical supervision was made available prior to the study, during it, and for 3 days afterwards. During medical examinations, no health contraindications to participation in the experiment were found. Additionally, participants were required to make a declaration that, for at least one month before the period of the study, they had not taken any medications or dietary supplements, and had not engaged in any form of smoking or the drinking of alcohol. Moreover, participants declared that they had not participated in fasting for longer than 3 days in the last 6 months. Before commencing the study, the participants were fully informed about the study’s procedures, and possible risks. Prior written consent to participate in the study was obtained from each participant. The experimental protocol was approved by the Research Ethics Committee of the Jan Długosz University in Częstochowa, Poland (KE-0/1/2019). All procedures performed in studies was conducted in accordance with the Declaration of Helsinki.

### Study protocol

The subjects of the study came to the laboratory in the morning of the first day, between 8:00 a.m. and 9:30 a.m., after having fasted for 12 h. Age was noted, and somatic data (body height—BH, body weight—BM, fat tissue content—BF, fat-free mass—FFM, total body water—TBW, and body mass index—BMI) were recorded, using the Tanita TBF 300A body composition analyzer (Tanita, Amsterdam, the Netherlands). Then, with the subject sitting down, blood samples were taken from the antecubital vein. The blood was allowed to clot at room temperature and then centrifuged at 1500×g for 15 min. The serum so obtained was divided into aliquots and stored at − 80 °C for later analysis. Additional blood samples were collected in heparinized tubes. Plasma was also obtained from the blood by treating it with EDTA. Then, the concentrations of: glucose (G), triglycerides (TG), free fatty acids (FFA), glycerol (GLY), β-hydroxybutyrate (β-HB), total protein (TP), insulin-like growth factor binding protein (IGF-BP3), urea (U) and uric acid (UA) were determined.

Concentrations of hormones such as insulin (IRI), insulin-like growth factor 1 (IGF-1), insulin-like growth factor 2 (IGF-2), growth hormone (GH), cortisol (C), leptin (L), ghrelin (GHRL), orexin A (ORE-A), orexin B (ORE-B) were also determined. Additionally, the concentrations of total antioxidant status/capacity (TAS/TAC), total oxidative status/capacity (TOS/TOC), interleukin IL-6, and human tumor necrosis factor alpha (TNF-α) were determined in the serum. A capillary blood sample was also collected to determine the lactate concentration (LA). The homeostatic model assessment of insulin resistance (HOMA-IR) was defined as fasting serum insulin (milliunit per liter) multiplied by fasting glucose (millimole per liter) divided by 22.5.

Then, a cycloergometric examination of the lower limbs was performed. This test started at 60 W and the load was increased by 30 W every 3 min until the individual maximum exercise capacity was achieved. The work rate was constant and amounted to 60 rpm. Immediately after the end of the exercise, venous blood was collected again and the concentrations of the above-mentioned biochemical variables were determined according to the procedure described above. On the ninth morning, the subjects returned to the laboratory, where the research procedure used before the 8-days of water-only fasting was repeated.

### Study procedure

Spinreact (Girona, Spain) tests were used to determine the concentration of the following metabolites: glucose (GOD-POD, liquid test), triglycerides (GPO-POD, liquid test), total protein (Biuret Colorimetric), urea (Urease-GLDH, liquid test), uric acid (Uricase-POD, liquid test). Moreover, β-hydroxybutyrate concentration was determined enzymatically using the RANBUD diagnostic kit (Randox Laboratories Ltd., Crumlin, County Antrim, UK). Among the metabolites analysed, serum FFA and glycerol concentrations were determined using colorimetric/fluorimetric assay kits (EFFA-100 and cat. no. EGLY-200 respectively) (BioAssay Systems, Hayward, California). Capillary blood lactate concentration was determined using the Epoc Blood Analysis System (Ottawa, Canada). Serum insulin concentration was determined by the immunoradiometric method using Immunotech SA kits (France). Human ELISA kits (Bioassay Technology Laboratory, China) were used to determine the levels of: GHRL (Cat. No. E3091Hu), ORE-A (Cat. No. E1296Hu) and ORE-B (Cat. No. E1282Hu). The CLIA method was used to determine the levels of GH, IGF-1, IGF-BP3, IL-6, and cortisol in serum using the MAGLUMI -1000 apparatus with appropriate kits (Snibe Diagnostic, Shanghai International Holding Corp. GmbH, Europe, Hamburg, Germany; Cortisol-CLIA, Shenzhen New Industries Biomedical Engineering Co. Ltd., Shenzhen, China). An immunological kit from DRG International, Inc., USA was used for the quantitative determination of leptin concentration in serum—DRG Leptin (Sandwich) ELISA (EIA-2395). The concentration of IGF-2 in serum was determined enzymatically using an ELISA immunoassay kit (Mediagnost GmbH, Tübingen-Reutlingen, Germany). Serum total antioxidant status/capacity (TAS/TAC) was determined using the Immundiagnostik test (Immundiagnostik AG, Bensheim, Germany). The PerOx (TOS/TOC) photometric test kit was used to determine the total oxidative state/capacity (TOS/TOC) in serum (Immundiagnostik AG, Bensheim, Germany). TNF-α concentration was determined using DRG immunoenzymetric assay (EIA-4641), (DRG International, Inc., Springfield, New Jersey, USA).

### Statistical analysis

Mean (M) and standard deviation (± SD) values were calculated for each parameter. The Shapiro–Wilk test was used to check the normality of distribution. A paired t-test was used to determine the effect of 8-days of water-only fasting on somatic variables. Nonparametric Friedmann repeated-measures ANOVA by rank was also used, utilising the Dunn-Bonferroni post hoc test to determine the effects of fasting and exercise testing. Partial eta-squared (ηp^2^) was calculated to determine effect size. Values at *p* < 0.05 were considered statistically significant. The values obtained were subjected to statistical analysis using SPSS Statistics 20. Sample size analysis was performed using Statistica v 13.3 (TIBCO) based on the results of the pilot study. We estimated the sample size necessary to demonstrate statistical significance between the study groups *p* = 0.05 and a test power of 0.8, which was 12 patients.

## Results

In terms of somatic changes, a significant reduction in BW, relative (%) and absolute (kg) BF values, absolute (kg) FFM and TBW values as well as BMI (*p* < 0.001) were observed under the influence of 8-days of water-only fasting. Moreover fasting intervention increased relative (%) values of FFM (*p* < 0.01) and TBW (*p* < 0.001).

The results presented in Table 1 show that carbohydrate metabolism was significantly modified under the influence of 8-days of water-only fasting and exercise tests (*p* < 0.001).

**Table 1:** Changes in the carbohydrate metabolism (M ± SD) after 8-days of water-only fasting and two physical exercise tests.

Values	Rest before fasting (1)	Rest after fasting (2)	Exercise before fasting (3)	Exercise after fasting (4)	Friedman’s Anova; *p* ≤	Partial eta square ηp^2^
G [mmol/L]	4.96 ± 0.54	3.80 ± 0.57**	5.92 ± 0.75^##^	4.46 ± 0.67	0.000001	0.114 (M)
IRI [mU/L]	6.32 ± 1.38	4.86 ± 1.57	6.32 ± 0.88^###^	3.89 ± 1.56	0.00003	0.081 (M)
HOMA-IR [mU/mmol]	1.39 ± 0.34	0.82 ± 0.30**	1.66 ± 0.40^##^	0.77 ± 0.41	0.000001	0.109 (M)
LA [mmol/L]	1.29 ± 0.63	1.67 ± 0.54^###^	8.53 ± 2.94***	6.85 ± 1.81	0.000001	0.356 (L)

Dunn-Bonferroni comparisons: different than (1) *p < 0.05; **p < 0.01; ***p < 0.001; different than (4) ^##^*p* < 0.01, ^###^*p* < 0.001. Effect size: ηp^2^ = 0.01 indicates a small effect (S); ηp^2^ = 0.06 indicates a medium effect (M); and ηp^2^ = 0.14 indicates a large effect (L).

Fasting significantly reduced G concentrations and HOMA-IR values both at rest and after exercise. However, only the combination of fasting and physical activity significantly lowered insulin levels. Fasting did not affect lactate concentration, which significantly increased (*p* < 0.001) after physical exercise, regardless of the fasting intervention.

The results in Table 2 show that variables related to fat and protein metabolism (FFA, GLY, TG, β-HB, TP, and UA) also changed significantly under the influence of the fasting and exercise tests. The fasting caused a significant increase in the concentration of β-HB and UA, both at rest and after exercise. The fasting also increased the concentration of FFA (*p* < 0.01) and GLY (*p* < 0.05) in serum under resting conditions. It should also be noted that the exercise test increased GLY (*p* < 0.01) and TP (*p* < 0.001) levels before fasting, as well as TG (*p* < 0.05) and TP (*p* < 0.001) concentrations after fasting. Fasting did not appear to impact the TP, which was significantly influenced by exercise.

**Table 2:** Variables related to fat metabolism, ketosis, and protein metabolism (M ± SD) after 8-days of water-only fasting and two physical exercise tests.

Values	Rest before fasting (1)	Rest after fasting (2)	Exercise before fasting (3)	Exercise after fasting (4)	Friedman’s Anova; *p*≤	Partial eta square ηp^2^
FFA [µmol/l]	220.55 ± 84.75	303.10 ± 122.53**	213.22 ± 62.28	251.66 ± 92.05	0.00216	0.019 (S)
GLY[mmol/l]	0.23 ± 0.07	0.30 ± 0.09*	0.33 ± 0.13**	0.43 ± 0.13	0.00001	0.089 (M)
TG [mmol/l]	90.23 ± 33.75	112.54 ± 28.1^#^	105.46 ± 36.68	136.23 ± 27.17	0.0001	0.056 (S)
β-HB[mmol/l]	0.31 ± 0.21	4.55 ± 1.04***	0.21 ± 0.16^###^	3.86 ± 1.06	0.000001	0.528 (L)
TP [g/L]	74.33 ± 4.14	74.60 ± 4.47^###^	81.54 ± 3.91***	81.82 ± 4.55	0.000001	0.110 (M)
U [mg/dl]	28.63 ± 5.05	32.05 ± 9.30	29.12 ± 4.83	32.81 ± 7.86	0.18964	0.012 (S)
UA [mg/dl]	6.56 ± 0.93	13.80 ± 2.48**	6.86 ± 0.96^###^	11.40 ± 2.49	0.000001	0.309 (L)

Dunn-Bonferroni comparisons: different than (1) *p < 0.05; **p < 0.01; ***p < 0.001; different than (4) ^###^*p* < 0.001. Effect size: ηp^2^ = 0.01 indicates a small effect (S); ηp^2^ = 0.06 indicates a medium effect (M); and ηp^2^ = 0.14 indicates a large effect (L).

As shown in Table 3, in terms of hormonal changes, the study showed that 8-days of water-only fasting and exercise tests caused significant changes in the concentrations of IGF-1, IGF-BP3, GH, L, and C, ORE-B and IGF-2. Post hoc analysis showed that fasting changed the concentrations of IGF-1, IGF-BP3, L and C at rest and under post-exercise conditions, as well as IGF-2 only at rest. Physical exercise resulted in a significant increase in IGF-BP3 concentration (*p* < 0.01) before fasting intervention. Additionally, exercise significantly enhanced GH concentration (*p* < 0.01). In summary fasting alone produces a statistically significant reduction in IGF-1, IGF-2, IGF-BP3, and L, while simultaneously elevating levels of GH and C. Notably, incorporating physical activity amplifies the positive effects of fasting on GH.

**Table 3:** Hormonal changes in the serum (M ± SD) after 8-days of water-only fasting and two physical exercise tests.

Values	Rest before fasting (1)	Rest after fasting (2)	Exercise before fasting (3)	Exercise after fasting (4)	Friedman’s Anova;* p*≤	Partial eta square ηp^2^
IGF-1 [ng/ml]	120.95 ± 32.84	65.21 ± 22.65**	133.72 ± 39.73^##^	73.06 ± 25.78	0.000001	0.108 (M)
IGF-2 [ng/ml]	1.65 ± 0.27	1.41 ± 0.18*	1.62 ± 0.19	1.58 ± 0.42	0.01430	0.016 (S)
IGF-BP3 [ng/mlµ]	2.91 ± 0.69	2.23 ± 0.65**	3.26 ± 0.63**^; #^	2.53 ± 0.57	0.00001	0.036 (S)
GH [ng/l]	1.47 ± 0.69	2.54 ± 1.43^##^	4.14 ± 3.68^#^	11.22 ± 5.57	0.00001	0.155 (L)
GHRL [ng/l]	1.55 ± 2.34	1.86 ± 2.77	1.69 ± 2.38	1.89 ± 2.70	0.05937	0.001 (S)
L [ng/ml]	2.21 ± 1.28	1.46 ± 0.34***	2.37 ± 1.45^##^	1.49 ± 0.33	0.000001	0.023 (S)
ORE-A [pg/ml]	346.68 ± 478.29	407.91 ± 537.26	379.75 ± 481.22	414.11 ± 497.12	0.39163	0.001 (S)
ORE-B [ng/L]	151.87 ± 190.63	163.65 ± 194.44	167.23 ± 217.18	179.58 ± 217.14	0.00609	0.001 (S)
C [ng/ml]	304.93 ± 89.81	499.05 ± 138.41*	309.78 ± 116.83^###^	515.41 ± 159.44	0.000016	0.093 (M)

Dunn-Bonferroni comparisons: different than (1) *p < 0.05; **p < 0.01; ***p < 0.001; different than (4) ^#^*p* < 0.05, ^##^*p* < 0.01, ^###^*p* < 0.001. Effect size: ηp^2^ = 0.01 indicates a small effect (S); ηp^2^ = 0.06 indicates a medium effect (M); and ηp^2^ = 0.14 indicates a large effect (L).

As shown in Table 4, variables determining the oxidative status and body immunity, i.e. TAS, PerOX, and IL-6, also changed significantly as a result of the combined effect of fasting and physical exercise. Trends were observed showing increased TAS concentration and reduced PerOx after fasting. Additionally, exercise significantly enhanced the effects of fasting for both parameters. However, the exercise test induced an increase in IL-6 concentration (*p* < 0.001) after 8-days of water-only fasting. In summary, fasting alone does not lead to statistically significant changes in oxidative stress and IL-6, whereas the combination of fasting and physical activity does introduce changes in these markers.

**Table 4:** Oxidative/antioxidant status and cytokine changes (M ± SD) after 8-days of water-only fasting and two physical exercise tests.

Values	Rest before fasting (1)	Rest after fasting (2)	Exercise before fasting (3)	Exercise after fasting (4)	Friedman’s Anova; *p*≤	Partial eta square ηp^2^
TAS [µmol/l]	304.40 ± 35.91	335.72 ± 37.23	315.92 ± 34.22^#^	352.91 ± 25.82	0.00363	0.060 (M)
PerOx [µmol/l]	227.04 ± 122.23	111.55 ± 108.36	297.63 ± 175.33^##^	158.61 ± 207.18	0.00039	0.019 (S)
IL-6 [pg/ml]	4.87 ± 1.96	4.162 ± 1.02^###^	5.69 ± 1.58	5.96 ± 1.90	0.00045	0.018 (S)
TNF-α [pg/ml]	3.71 ± 1.54	4.37 ± 1.96	3.44 ± 2.10	4.35 ± 2.91	0.66892	0.004 (S)

Dunn-Bonferroni comparisons: different than (4) ^#^*p* < 0.05, ^##^*p* < 0.01, ^###^*p* < 0.001. Effect size: ηp^2^ = 0.01 indicates a small effect (S); ηp^2^ = 0.06 indicates a medium effect (M); and ηp^2^ = 0.14 indicates a large effect (L).

## Discussion

In the current study the combined effect of 8-days of water-only fasting and an exercise test performed twice resulted in a significant reduction in glucose, insulin and HOMA-IR. Moreover, the fasting element induced a greater share of these changes than the two sets of exercise. Numerous studies indicate that reducing the level of glucose in the bloodstream leads to a decline in the concentration of circulating insulin while increasing the concentration of glucagon, even on the first day of fasting^23^. This reduced amount of glucose in the blood is nonetheless sufficient to meet the glycolytic needs of the brain and other tissues during the initial days of fasting^24^. However, after 8-days of water- only fasting, the body’s metabolism undergoes significant changes. These changes are intense and extended over time, and already after 24 h of fasting glycogen stores are depleted and the body begins to produce glucose through the process of gluconeogenesis in the liver. Despite reduced levels, insulin continues to moderate the rate of gluconeogenesis by promoting lipolysis and providing the liver with fatty acids^25^. At the same time, the increased concentration of glucagon in this phase of fasting causes a decrease in the level of malonyl-CoA. This leads to the formation of ketone bodies, which the brain uses instead of glucose. This helps to conserve glucose, which is used by many tissues, including red blood cells, bone marrow and kidney medulla. Then prolonged fasting shifts the gluconeogenesis process from the liver to the kidneys^26^. Low glucose and insulin concentrations caused by fasting are believed to decrease the risk of cardiovascular disease (CVD)^27^ and to help the body adapt to fasting conditions^28^. In the current study, prior to the 8-day water-only fast, normal levels of glucose and insulin concentrations, as well as correct values of the HOMA-IR index, were observed. However, a significant decrease in these values after 8 days of water-only fasting suggests that it was this factor that controlled glycemia at that time^29^.

In the current study, as well as in previous works^7,27^, there was no significant effect of single maximum intensity physical exercises on glycemic control. After cycloergometric exercise test it was observed that lactate concentration had significantly increased, compared to resting conditions, as measured both before the 8-days of water-only fasting and at its completion. These results suggest that glycolysis plays an important role in providing energy protection to the body, even when blood glucose concentrations are reduced.

Moreover, under these conditions, lactate inhibits lipolysis and limits the mitochondrial uptake of fatty acids by muscles^30^. Despite the moderate impact of a single exercise on metabolism, it should be borne in mind that repeated physical exercise, included in the training framework, is important in the control of glycemia and insulin resistance^31^ and in health prevention^32^.

The combined effect of fasting and physical exercise caused significant changes in body weight, including a reduction in body fat. Such a change can be associated with the fact that from the second day of fasting there is a significant increase in the concentration of FFA and glycerol in the blood, which indicates the stimulation of lipolysis^1,33^. In the current study, 8-days of water—only fasting resulted in further intensification of lipolysis, as both serum FFA and glycerol concentrations remained elevated, although triglyceride concentrations did not change at rest. It was also noticed that already in the initial days of fasting, increased concentrations of FFA and glycerol in plasma were accompanied by an increase in the concentration of gluco- and ketogenic amino acids^23^. Together, these changes activate the process of gluconeogenesis and ketogenesis in the liver. The plasma concentration of β-HB, the most important ketone body, increased many times in our study and slightly exceeded the upper limit of ketonemia^34^. This condition can cause ketoacidosis and may be dangerous to a person’s health. It has also been noted that increased concentrations of ketone bodies during fasting may be beneficial for the body because these substrates are used by the brain, skeletal muscles, kidneys and heart^23^.

Therefore, because of possible ketoacidosis, it should be acknowledged that an 8-days of water-only fasting, despite its advantages, may pose a health risk, especially to individuals with liver disease of any kind^35^.

However, these changes were not reflected in the concentration of total protein in the serum of the men we examined, because its concentration did not change, which may also be related to the described effect of saving body proteins in the later days of fasting^1^. It also appears that the increased release of glucogenic and ketogenic amino acids described above^23^ did not affect changes in serum total protein concentrations.

The lack of change in the concentration of total protein in serum is confirmed by the unchanged concentration of urea after the fasting intervention. Moreover, a significant increase in total protein concentration was observed after exercise tests, both before and after the fasting intervention^17,36^. However, the lack of changes in post-exercise urea concentration indicates that this increase was not caused by metabolic changes in total protein and was probably related to, among others, a transient decrease in plasma volume in the post-exercise period^37^.

The 8-days of water-only fasting resulted in a more than two-fold increase in serum uric acid concentration, and the physical exercise did not modify this change. On the one hand, an increase in this concentration can be considered beneficial because uric acid acts as a strong antioxidant and accounts for approximately 60% of the antioxidant potential of human plasma^38^. However, it is known that in the cellular environment this compound increases oxidative stress, which can cause systemic inflammation^39^. It has also been shown that hyperuricemia causes significant changes in systemic and renal hemodynamics, which may result in loss of autoregulatory renal function^40^ or lead to hypertension, insulin resistance, obesity, hypertriglyceridemia and metabolic syndrome^41^. Observation of the volunteers we studied several days after the end of the famine did not reveal any of these negative effects.

Observations of volunteers participating in our experiment, which we conducted for several days after the end of the starvation, did not show any of these negative effects.

The described experiment, which included fasting intervention and two exercise tests, took place under conditions of severe stress, which, among other things, activates the hypothalamic–pituitary–adrenal (HPA) axis. To monitor HPA activity, the study measured serum cortisol concentrations, which increased significantly after 8 days of water-only fasting, both at rest and after exercise. Also, the study by Steinhauser et al.^33^ showed a significant increase in cortisol levels already on the first day of fasting. Moreover, these studies showed a negative correlation between serum cortisol levels and the duration of caloric restriction. This means that the concentration of cortisol in the serum, as an important stimulator of lipolysis, increases in the initial days of caloric restriction, then decreases significantly and returns to the initial level after a few weeks of fasting. The 8-days of water-only fasting undertaken in the current study was long enough to maintain cortisol concentration at a much higher level than the baseline. Our previously unpublished data showed that 14-day and 21-day fasting resulted in further increases in serum cortisol concentrations. It appears, therefore, that elevated serum cortisol concentrations persist for longer than 3 weeks in cases of complete fasting. Physical exercise is a factor that increases plasma cortisol concentration^42^. However, in the current study, exercises testing did not increase serum cortisol concentrations either before or after fasting. Therefore, the physical exercise used was too weak a factor in stimulating cortisol secretion, while 8-days of water only-fasting showed such an effect.

An important aspect of various forms of caloric restriction is their impact on the feeling of hunger and activation of hypothalamic circuits regulating appetite. While leptin inhibits appetite^43^, orexigenic peptides such as GHRL and orexins enhance this feeling and activate the hunger center^44^. The current study shows that 8-days of water-only fasting definitely led to hypoleptinemia, while the concentrations of GHRL, ORE-A, and ORE-B did not change significantly under these conditions, and only increasing trends of these hormones appeared. The occurrence of hypoleptinemia and no or slight increases in GHRL concentration had previously been observed after 72 h of fasting in people with diabetes^45^, after 4 days of caloric restriction in adult men^43^, or after 1 week of intermittent Ramadan fasting^46^. The study by Steinhauser et al.^33^ suggested a possible correlation between decreased leptin levels and increased cortisol concentrations during fasting, which also seems likely in the current study. Other authors also indicated that acute fasting-induced hypoleptinemia occurs already on the first day of fasting and may stimulate the HPA axis and thus cause a switch from carbohydrate metabolism to lipid metabolism in humans. Hypoleptinemia and nosignificant changes in GHRL and orexins concentrations occurring in the current study indicate that after 8-days of water-only fasting there is no acute activation of the hunger center and no significant increase in appetite. This observation is confirmed by the statements of people who have fasted, indicating that the strongest feeling of food craving occurs on the first day of fasting, after which it gradually decreases. Scientific reports also indicate the excellent mental well-being of volunteers subjected to 8-days of water-only fasting^47^. The physical exercise tests used in the current study did not cause any changes in the concentrations of leptin, GHRL, ORE-A, and ORE-B.

The study of Hollstein et al.^48^ indicated that an important issue in energy adaptation to fasting seems to be the activation of the GHRL/GH/IGF-1 axis. In the current study, the combined effects of fasting and physical exercise altered the concentrations of GH and IGF-1, with no impact on GHRL. Notably, there was only an upward trend observed in GH levels during resting conditions after the fasting intervention. This lack of change in GH concentration during rest contrasts with findings from other studies, which indicated an increase in this hormone following short-term fasting^49,50^. All these suggest that the separate effect of fasting or physical exercise is too weak a stimulus for changes in GH concentration. GH is known to achieve most of its anabolic effects by stimulating the release of IGF-1 by the liver. The study of Hollstein et al.^48^ showed a large increase in GH concentration during short-term fasting and no changes in IGF-1 concentration. In the current study, as already mentioned, there was an increase in GH concentration caused by fasting only under conditions of physical exercise, while the concentration of IGF-1 decreased significantly during fasting, both at rest and during physical exercise. The described negative correlations between GH and IGF-1^48^ suggest that IGF-1 may influence the secretion of GH by the hypothalamus using negative feedback^51^. The results of our study seem to confirm this relationship.

In the present study, IGF-BP3 and IGF-2 concentrations changed under fasting and exercise conditions^52^, and the exclusive effect of fasting reduced IGF-BP3 concentrations both at rest and during exercise. However, in another study, which included a 36-h fast, a decrease in IGF-1 concentration was observed in a group of healthy women without changes in IGF-BP3 concentration^50^. The parallel decrease in IGF-1 and IGF-BP3 concentrations during fasting in our experiment suggests that the intensity of the IGF-1 binding process by IGF-BP3 was limited compared to the state before fasting and that physical exercise had no effect on these relationships. IGF-1, like IGF-2, is structurally similar to insulin^53^. IGF-2 has a hypoglycemic effect, which is strongly manifested in some diseases^54^. In healthy men participating in our study, the glycemic effect of IGF-2 did not occur under the fasting intervention, because the concentration of this hormone decreased similarly to the concentration of glucose.

It is unclear whether and how some forms of fasting affect the immune system, despite their growing popularity due to their potential immunomodulatory effects^55^. For this reason, a serum concentration of IL-6 and TNF-α was examined in this study as a markers of host immune response. Although IL-6 is generally considered a pro-inflammatory molecule, numerous studies have shown that IL-6 can act as an anti-inflammatory cytokine^56,57^. Moreover, contracting skeletal muscle produces IL-6, which acts as a myokine^58^ and regulates multiple processes such as energy metabolism^16^, muscle regeneration^59^,bone remodeling^60,61^, and hormone secretion of hormones such as insulin^62^ and cortisol^63^. Furthermore, physical activity boosts IL-6 secretion, which is dependent on duration, intensity, and the size of muscle mass engagement^16^. In the current study, an increase in IL-6 levels was observed after the combined impact of fasting and exercise, as well as an increase in circulating FFA concentrations after fasting. It is likely that, in both conditions, the body switches from using glucose to utilizing fat as an energy source. All that leads to an upregulation of lipolysis, and an increased level of IL-6 in the blood may have a significant impact on this particular process^18^. In a separate study, it was demonstrated that monocytes were not a primary source of increased IL-6 following physical activity^64^. These results suggest that changes in the level of IL-6 were related more to its metabolic role as a myokine than to its immune function. Regardless of that possibility, further studies are necessary to comprehend how IL-6 signaling maintains homeostasis during fasting accompanied by physical activity.

It is currently unclear whether different types of fasting can substantially reduce the levels of key-proinflammatory cytokines such as TNF-α^19^. The fluctuation of TNF-α concentration during fasting depends both on the participants’ physiological condition, e.g. whether they are healthy, obese, physically active or not, and on other factors, such as the length of the fast, and the types of food restriction protocol in use^65^. While studies have demonstrated the benefits of intermittent fasting (IF) in reducing basal levels of TNF-α^66^, very little is known about the impact of zero-calorie fasting on immune defense. Surprisingly, in contrast to IF interventions, a 10-day zero-calorie diet increased systemic inflammation biomarkers such as IL-6 and TNF-α in circulation and subcutaneous adipose tissue. It also led to an increase in adipose tissue macrophages and enhanced systemic pro-inflammatory pathways. According to the authors of those papers, severe calorie restriction led to inflammation signals, which caused a metabolic shift towards lipid utilization. In this context, activated immune cells could help to clear up lipids, lipid by-products, and dying cells^67^. Results from the current study revealed that 8-days of water-only fasting and physical activity have no statistically significant effect on serum levels of pro-inflammatory TNF-α. However, there was an upward trend in the concentration of this cytokine after the fasting period. It is thus not excluded that prolonged calorie restriction could drive inflammation. Taken together, the results from the current study complement the existing data on the impact of water-only fasting on systemic inflammatory mediators. Nonetheless, further research is needed to determine the mechanisms underlying the immunomodulatory effects of a water-only diet.

Total antioxidant status (TAS) in serum represents the body’s ability to defend itself against oxidation. The current study found that an 8-days of water-only fasting improved blood TAS and reduced lipid peroxidation. This result is consistent with previous research that involved a 10-day fasting period, which resulted in decreased lipid peroxidation and increased total antioxidant capacity in the plasma^68^. Moreover, multiple studies have demonstrated that IF can reduce oxidative stress^69,70^. Also, regular physical exercise training is known as one of the most powerful ways to boost antioxidant response^71,72^. In the current study it was shown that fasting enhances the positive impact of physical activity on the body’s antioxidant status. Additionally, uric acid functions as a powerful scavenger of free radicals in plasma^73^ and its concentration correlates with increased antioxidant capacity during fasting^69,74^. Our study also shows that calorie restriction leads to elevated levels of uric acid, thus confirming the beneficial effects of water-only fasting on the body’s antioxidant status.

This paper highlights the positive aspects of water-only fasting, such as increased lipolysis and enhanced antioxidant reserves. However, it is important to summarize several potential risks associated with fasting. As previously noted, fasting raises the concentration of ketone bodies, which can result in ketoacidosis—a condition that poses significant risks for individuals with liver damage^32^. Additionally, dietary interventions that elevate uric acid levels may lead to hyperuricemia, negatively affecting hemodynamics, hypertension, and renal function^37,38^. During fasting, the body breaks down proteins, converting the released amino acids into substrates for gluconeogenesis^1,72^. This process has raised concerns about potential weakening of the muscular system. However, recent evidence compellingly demonstrates that after seven days of fasting, muscle strength and oxidative enzyme levels in skeletal muscle remain remarkably preserved. It is important to note, though, that this extended fasting period does lead to a decline in high-intensity endurance capacity^73^. This insight challenges the traditional view of fasting’s impact on muscle health and highlights the body’s remarkable adaptability. Recent research has revealed a fascinating phenomenon: during periods of fasting, monocytes are known to re-enter the bone marrow. This process has significant implications, as refeeding leads to increase in monocyte counts in the bloodstream, but also alters the body’s immune responses to bacterial infections^74^. Our research has demonstrated that the key pro-inflammatory cytokine IL-6 functions more like myokine and is linked to physical activity during fasting. Changes in myokines that occur during physical activity play a significant role in non-pharmacological therapies aimed at reducing obesity-related disorders^75^. We hypothesize that incorporating physical activity into fasting may influence the immune system, although we do not yet have direct evidence to support this. Understanding these interactions could revolutionize our approach to nutrition, fasting, and immune health.

## Limitations and future prospects

The presented study has several limitations. The main one is the relatively small group of 13 volunteers participating in them. It should be mentioned here that a group considered by some critics to be small in such a difficult experience as 8 days of complete fasting and the accompanying physical exercise of maximum intensity is extremely difficult to assemble and has not been the subject of research so far, perhaps due to such obstacles. Another limiting factor in this work is the discrepancy between the research protocols used by other authors and ours. Various forms of intermittent fasting or caloric restriction excluding physical exercise are the most frequently used in experiments. Another factor limiting drawing general conclusions is the daily variability of the concentrations of some hormones and the lack of examination of the long-term effects caused by 8-day fasting in the body. Although we avoided the first restriction by conducting research at a similar time of the day, in order to draw far-reaching conclusions, biochemical determinations would have to be made at different times of the day and several days and weeks after the end of the research. It also seems important to examine the impact of different types of physical exercise of varying intensity combined with the fasting intervention. Finally, the experimental protocol can also be improved by testing whether lifestyle influences the results, for example by dividing subjects into physically active and inactive groups or by consuming different types of diets before the experiment. We also failed to find similar studies conducted on women, both premenopausal women, characterized by high hormonal variability, and menopausal women.

Therefore, the presented research should be treated as preliminary, because diversifying the protocols of future research with the limiting factors described above would provide a broader picture of the impact of fasting combined with physical exercise on the human body.

## Conclusions

The results of this study showed that 8-days of water-only fasting had a broader impact on metabolic, hormonal, and immune changes than two sets of intense physical exercise in healthy middle-aged men. Significant metabolic changes involving reduction of carbohydrate metabolism and activation of fat metabolism, along with stimulation of ketogenesis, supported by improvement of antioxidant status and IL-6 signalling, appear to be beneficial because they lead to improved metabolic control of the body. The findings regarding the combination of fasting with physical activity, although preliminary, show promise as a useful tool for everyday life, such as during training or for weight maintenance. The results indicate potential health benefits related to changes in carbohydrate and lipid metabolism, which could be significant in the treatment of obesity and other metabolic diseases. Future research should aim to increase the study group size, evaluate the long-term metabolic and hormonal effects of prolonged fasting in comparison to a ketogenic diet, and implement the diet and exercise regimen in individuals with metabolic diseases.

## Data Availability

The datasets generated during the current study are available from the corresponding author upon reasonable request.
